# Intervention Fidelity Focusing on Interaction between Participants and Facilitators in a Telephone-Delivered Health Coaching Intervention for the Prevention and Management of Type 2 Diabetes

**DOI:** 10.3390/nu13113862

**Published:** 2021-10-28

**Authors:** Linda Timm, Ida Karlsson, Kristi Sidney Annerstedt, Pilvikki Absetz, Birger C. Forsberg, Meena Daivadanam, Helle Mølsted Alvesson

**Affiliations:** 1 Department of Neurobiology, Care Sciences and Society, Karolinska Institutet, 141 83 Huddinge, Sweden; 2 Department of Global Public Health, Karolinska Institutet, 171 77 Stockholm, Sweden; ida.karlsson.1@ki.se (I.K.); kristi.sidney@ki.se (K.S.A.); birger.forsberg@ki.se (B.C.F.); meena.daivadanam@kbh.uu.se (M.D.); Helle.Molsted-Alvesson@ki.se (H.M.A.); 3 Institute of Environmental Medicine, Karolinska Institutet, 171 77 Stockholm, Sweden; 4 Collaborative Care Systems Finland, 33500 Tampere, Finland; pilvikki.absetz@gmail.com; 5 International Maternal and Child Health Division, Department of Women’s and Children’s Health, Uppsala University, 751 85 Uppsala, Sweden; 6 Department of Food Studies, Nutrition and Dietetics, Uppsala University, 751 22 Uppsala, Sweden

**Keywords:** Type 2 Diabetes, prediabetes, lifestyle interventions, telephone support, self-management, implementation, interaction, fidelity, mixed methods

## Abstract

Self-management support and lifestyle interventions with an empowerment approach have been found to be effective strategies for health improvement among people at risk for or living with type 2 diabetes. Telephone coaching seems particularly efficient for individuals with low socioeconomic status and culturally and linguistically diverse backgrounds. In this mixed methods study, we investigate a telephone-delivered health coaching intervention provided by the diabetes project SMART2D (Self-Management Approach and Reciprocal learning for Type 2 Diabetes) implemented in socioeconomically disadvantaged areas in Stockholm, Sweden. We focus on the interaction between participants and facilitators as part of intervention fidelity. Recorded coaching sessions were scored using an interaction tool and analyzed by exploratory factor analysis and recorded supervisory discussions with facilitators analyzed using thematic analysis. The quantitative analysis showed that the intervention components were delivered as intended; however, differences between facilitators were found. The qualitative data highlighted differences between facilitators in the delivery, especially in relation to dietary and physical activity goalsetting. The level of language skills hindered the delivery flow and the tailoring of sessions to participants’ needs led to different delivery styles. The interaction between facilitators and participants is an important aspect of intervention implementation. Tailoring of interventions is necessary, and language-skilled facilitators are needed to minimize barriers in intervention delivery.

## 1. Introduction

The prevalence of T2D and diabetes risk is disproportionally distributed and shows a clear social gradient. Low socioeconomic status (SES) is an upstream determinant for developing T2D and its complications [1,2,3]. Lifestyle interventions and self-management support are effective strategies to manage and prevent type 2 diabetes (T2D) [4,5,6,7], as well as improve diabetes-specific quality of life [6]. This is especially important for prediabetes, a high-risk state with elevated glucose levels that is reversible and in which glucose levels can be normalized through lifestyle interventions [8,9]. Remission of T2D has also been reported as a result of intensive lifestyle modification interventions [10,11,12]. Yet uptake of interventions and their effectiveness are influenced by SES [13], pointing at access to lifestyle interventions being lower in low SES groups.

Telephone coaching as an intervention approach has shown to improve health behavior, self-efficacy and health status among persons with chronic conditions, in particular for individuals with low SES and culturally and linguistically diverse backgrounds, often with low access to health services [14]. The planned, unscripted telephone coaching was most suitable for this group of individuals, as it enabled health care providers to tailor the sessions to the participants’ needs [14]. A literature review on coaching interventions for chronic diseases [15] showed that it is important to consider the emotional state of the patient. As not all persons are ready to change their behavior, coaching interventions that move patients to a stage of action are useful [15]. The same review concluded that although the evidence base is insufficient at this time, coaching that does not involve face-to-face meetings, such as telephone-based coaching, may be as effective as face-to-face coaching [15].

Communication between the patient and health provider is central in health care situations but is often hindered by a lack of competence in intercultural communication skills, language barriers and differences in communication styles [16]. Understanding the patient’s world has been found to be more relevant than focusing on cultural differences [17,18], and T2D interventions with an empowerment approach have been found effective in improving clinical outcomes and reducing outpatient clinic utilization rates [19].

The empowerment approach suggested by Anderson and Funnel (2005) is a patient-centered process that encourages lead-taking in self-management through critical thinking and by acting autonomously to improve self-efficacy [20,21]. Empowerment is one of the dimensions in the Strength-based Behavior Coaching model, where identification of strengths and competencies leads to interaction that supports perceived autonomy, competence and relatedness, and enables building of behavior change by utilizing the identified strengths [22]. Empowerment is also a key component of motivational interviewing (MI) [23]. MI provides practical tools for helping clients/patients to explore and resolve ambivalence [23]. A central aspect of MI is the patient centeredness, where the provider creates appreciative collaboration with the patient. Interventions based on MI have been found to be particularly useful for clients who are reluctant to change or who are ambivalent about changing their behavior [23].

In addition to the type of approach, the interaction of actors (i.e., patients, caregivers and other involved stakeholders in networks) [24,25] is of importance for successful implementation of health interventions. Interaction in the patient–provider communication has a dual function of both information exchange and a relational function of interactions [26]. Although it can be argued that the responsibility in the interaction between providers and patients should rely on the providers due to the hierarchical positions [26], utilization of techniques such as MI and other empowerment approaches should lead to nonhierarchical, autonomy-supportive interaction that fosters a collaborative spirit. Collaborative health consultations are essential for outcomes such as patient satisfaction, self-management and adherence [27]. More research is needed on how to implement interventions, especially for hard-to-reach populations, and different aspects of fidelity need to be evaluated. In this study, we have focused on understanding dimensions of interaction as part of intervention fidelity.

Intervention fidelity is defined as “methodological strategies used to monitor and enhance reliability and validity of behavior interventions” [28]. A recent review concludes that fidelity needs to be seen as a multifaceted concept as it includes dimensions other than the intervention delivery by providers, such as training of the delivery and material used [29]. Moreover, different definitions and concepts have been used inconsistently in the previous literature about fidelity [29]. The balance between intervention fidelity and adaptation has been recognized as a dilemma, as the implementation of interventions to real-life settings often requires conducting changes to the original protocol [29]. For evaluating an intervention using a technique such as MI, assessing fidelity is important for knowing to what extent the potential improvements are a result of the approach and not from other components. However, research shows that MI as intervention strategy is often reported without evaluating the extent the technique is actually used, and therefore, criteria to check MI fidelity have been suggested [30,31]. Elements of the MI approach were integrated in the coaching strategy used to deliver the telephone-facilitated health coaching intervention investigated in this study.

The overall aim of this mixed methods study was to evaluate the intervention fidelity of a telephone-facilitated health coaching intervention to manage or prevent T2D by analyzing the interaction between facilitators and participants in relation to its dimensions, enablers and challenges.

## 2. Materials and Methods

This study was nested in SMART2D (Self-Management Approach and Reciprocal learning for Type 2 Diabetes) (ISRCTN 11913581), a 5-year project (2015–19) on implementation of contextualized T2D self-management support in Sweden, South Africa and Uganda [32]. We used a mixed methods research design conducted as part of the feasibility trial implemented in the Swedish arm of the SMART2D in socioeconomically disadvantaged areas of Stockholm [33]. Inclusion criteria for participation was to be registered at an address in one of the selected study sites. The participants for the Swedish arm were recruited through community screening arranged by the SMART2D project [34] and from registers at cooperating primary health care centers. A total of 265 persons with T2D or at high-risk of developing diabetes were included in the trial. Being at high risk was defined as either having prediabetes or a score of ≥13/25 points on the Finnish diabetes risk score (FINDRISC) [35]. The participants were cluster randomized into an intervention (*n* = 131) and a control group (*n* = 134).

### 2.1. Intervention

The Swedish SMART2D intervention consisted of nine structured telephone-facilitated support sessions focusing on lifestyle behaviors for each participant. Excluding the introduction and closing sessions, the remaining seven sessions focused alternately on diet and physical activity (Table 1). To be considered as having completed the intervention, a completion of ≥3/9 sessions was required. The completion rate was in line with agreed equal proportions for all SMART2D study sites [36].

Social support was encouraged throughout the program through care companions, defined as family members and/or friends who work together with participants in establishing goals and carrying out their health-related activities. In addition, general meetings were arranged at two time-points per study area for the participants to meet each other, the health coaching facilitators, SMART2D team members and representatives from the collaborating primary health care centers and citizen service offices. These meetings consisted of a lecture held by experts on diabetes, physical activity and diet and a question and answer session.

Initially peer group sessions were planned and piloted. The low interest in group counseling led to a change in the coaching approach toward individually delivered sessions by phone. This delay between the recruitment and start of the telephone-facilitated health coaching led to a loss of participants. A total of 72 persons started the intervention (T2D: 29; high-risk: 43), i.e., participated in the introductory session and the individually facilitated telephone coaching sessions, which were delivered over a 6-month period.

The majority of the sessions were delivered in either Swedish or English by four trained facilitators from the SMART2D team. The facilitators had a background in global and public health; three were female and one was male, with an average age of 36.5 years. The facilitators delivered all sessions to the participants individually, and each participant had their personal facilitator. Additionally, facilitators skilled in Somali, Arabic or Spanish delivered the sessions when needed. Training sessions were conducted for the facilitators prior to each intervention session in a roleplay format where they had the opportunity to practice the delivery of the intervention on each other followed by a discussion about the material and potential challenges. The median duration per session was 19 min (range: 12–25 min).

### 2.2. Study Participants

Study participants were the facilitators who delivered the sessions in either Swedish or English and the individuals who received the intervention. The individuals who received the intervention were asked for permission to record their health coaching sessions. The facilitators used the principle of maximum variation in identifying/selecting sessions to record based on the participant’s age, gender, T2D or prediabetes/high-risk status. Each of the facilitators recorded at least one of each coaching session, numbered one to nine. The four facilitators who delivered the majority of the intervention sessions in Swedish or English were invited to group discussions.

### 2.3. Study Design

**Figure 1:**
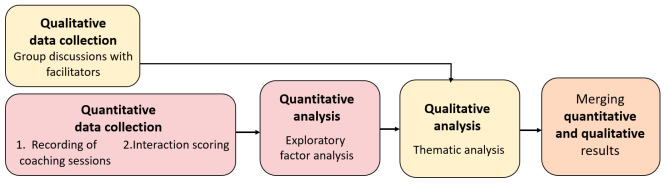
Mixed methods study process.

We used an explanatory sequential mixed methods study design [37] in which the quantitative analysis was conducted to inform the qualitative analysis (Figure 1).

The qualitative data from the facilitators and the quantitative data from the recorded sessions were collected in parallel during the intervention process. The scoring of recorded sessions was conducted after the intervention and the quantitative analysis before the qualitative analysis.

#### 2.3.1. Part 1: Quantitative Data Collection and Analysis

**Figure 2:**
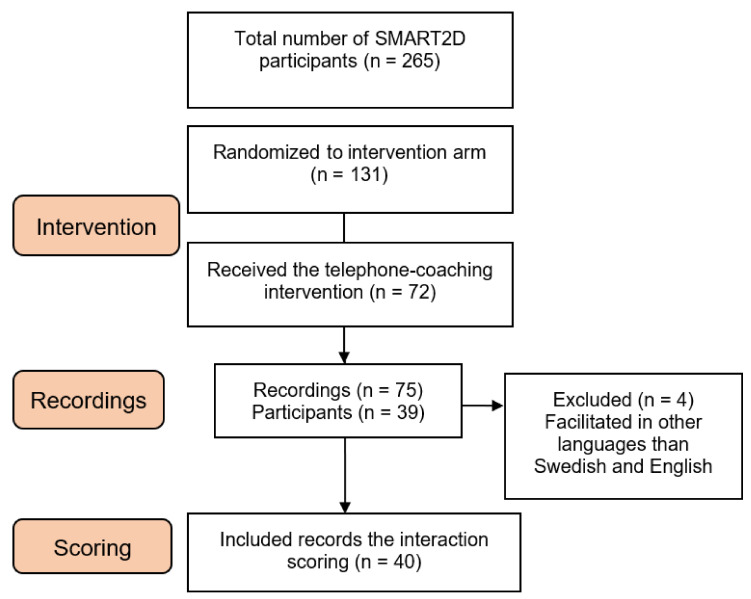
Flow chart describing sampling of SMART2D participants and recordings for the interaction scoring.

We used a longitudinal design in which intervention sessions were recorded and scored using a tool developed by the SMART2D consortium for this purpose. A total of 75 telephone-facilitated coaching calls from 39 individuals were recorded. After excluding four recordings in languages other than Swedish or English, a similar number of recordings for each of the nine sessions and from each of the facilitators were selected. A total of 40 sessions (three to six recordings from each of the nine sessions) were included in the interaction scoring and analysis (Figure 2).

#### 2.3.2. Interaction Scoring Tool

**Figure 3:**
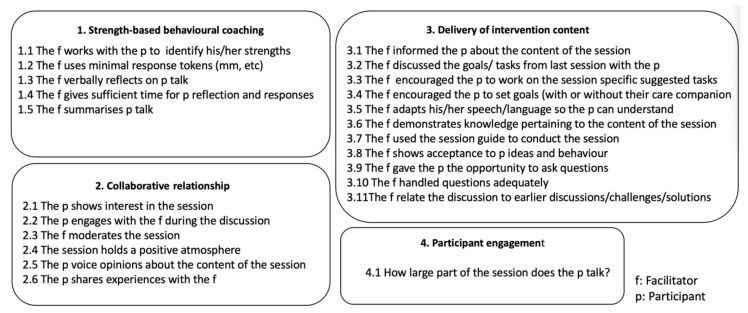
Statements and question included in the interaction scoring tool.

To evaluate the intervention fidelity, 40 selected recordings were scored using an interaction scoring tool consisting of 23 statements that assessed the interaction between the participant and facilitator based on four constructs: (1) Strength-based behavioral coaching techniques with focus on participants self-determination and strengths; (2) Collaborative relationship between facilitator and participant with focus on interest, atmosphere, moderation and experience; (3) Delivery of intervention content with focus on information, knowledge, goals and acceptance; (4) Participant engagement measured by the percentage of time the participant spoke in the session. The main focus was on the fidelity aspects of adherence and delivery. Figure 3 gives an overview of the statements included in the scoring tool.

#### 2.3.3. Scoring

The recordings used for the interaction scoring were selected using a random sequence generator, with emphasis on maximizing the range of participants and sessions. The sessions were rated independently by two SMART2D researchers who had not been involved with facilitating the intervention. The researchers have a background in epidemiology, public and global health. One is a nurse by training, and one is an occupational therapist. Standard operating procedures were created to maximize consistency in the evaluation and updated during the pilot phase, as well as during the scoring process when questions arose between the two raters. Interrater reliability (IRR) and Cohen’s kappa were calculated to control for similarities between the two raters [38]. A third rater was included to decide on the final scoring in sessions with differences larger than 50% among the two raters. The scoring was conducted using the data management tool REDCap [39]. The interaction tool was evaluated using exploratory factor analysis (EFA) to arrange the statements in the scoring tool and to report on the results according to the constructs. To control that the dataset was suitable for conducting EFA, Bartlett’s test of sphericity that indicates there are sufficient intercorrelations and the Keiser–Mayer–Olkin (KMO) measure of sampling adequacy were calculated [40]. Cronbach’s alpha was used to assess the internal consistency of the tool, with scores between 0.7 and 0.9 considered appropriate [41]. Likert summated scales were calculated to assess the items included in the factors from the EFA, in total and per facilitator.

The statements in constructs 1–3 were scored using a 5-point Likert scale ranging from strongly agree to strongly disagree. The question in construct 4 was answered as percentage of time the participant talked in the session, rated on a 5-point scale of 0–100% (Figure 3).

#### 2.3.4. Data Analysis

Four recordings were used to pilot test the tool, resulting in an IRR of 68%, 72%, 64% and 50%, with Cohen’s kappa of 0.57, 0.60, 0.44 and 0.19, respectively. The raters discussed differences in the rating and rated an additional two sessions, resulting in an IRR of 41% and 46%, with Cohen’s kappa of 0.04 and 0.17. Differences between the raters ranged from strongly agree to agree or from neutral to agree/disagree. After discussions, the standard operating procedures were updated and the remaining sessions rated. We considered an IRR above 50% (indicating moderate reliability) across all ratings to be sufficient for using an average score, since differences ranged only by one step of the Likert scale. The average score of the two raters was calculated for the recordings with an IRR >50% (24/40 recordings) and for the scorings with an IRR <50% (16/40 recordings) [38]; a third rater was included to decide on the final scoring of the differences.

For the EFA, three statements (3.2, 3.10 and 3.11) were excluded due to missing values. The Bartlett’s test of sphericity showed a *p*-value of 0.000 and a KMO of 0.635, indicating that the data were suitable for conducting EFA. Oblique rotation (promax) was conducted to identify constructs and eigenvalues. Parallel analysis was used to select the number of factors to be retained, and factors with eigenvalues >1 were kept. Items (statements) with loadings ≤0.4 or with cross-loadings ≤0.2 were dropped from the analysis. Four factors had eigenvalues >1 and were retained, and two statements (3.5 and 3.8) had factor loadings <0.4 and were removed. Statement 1.2 was reversed but did not function well either reversed or not and was therefore removed. The following EFA analysis had a KMO of 0.682 and three factors with eigenvalues >1. Cross-loadings were found on an additional two statements (2.3 and 2.4) that were also removed. The final analysis was conducted on 14 statements (1.1, 1.3, 1.4, 1.5, 2.1, 2.2, 2.4, 2.6, 3.1, 3.3, 3.4, 3.6, 3.7, 3.9) and had a KMO of 0.711.

The sum scores from the Likert summated scales were obtained by adding the scores from each item in the factor and subtracting the minimum possible score of those items (i.e., a construct with four items holds a sum score of x and a minimum score of 4 × 1 from the 5-point Likert scale = a sum score of 20–4). The sum score was standardized to range from 0 to 100 by multiplying the sum score—the minimum possible score—by 100 / m x (k −1), where m = # items and k = # Likert scale points [42]. Wilcoxon rank–sum test was conducted to detect differences between the sum score for each facilitator. Participant engagement was analyzed using descriptive statistics.

#### 2.3.5. Part 2: Qualitative Data Collection and Analysis

Four facilitators participated in four group discussions focusing on the enablers and challenges to intervention delivery during the intervention period, led by two moderators as part of quality assurance [43]. The meetings lasted between 1 and 1.5 h, and guides were used to cover different topics, such as preparation before coaching sessions, documentation during and after sessions, reflections on session content and supporting strategies. In addition, interaction between the facilitator and participant was discussed. The focus was on understanding the ways in which the facilitators interpreted engagement and how they encouraged or facilitated participants to take a lead during the coaching sessions. The meetings were recorded and transcribed verbatim.

**Figure 4:**
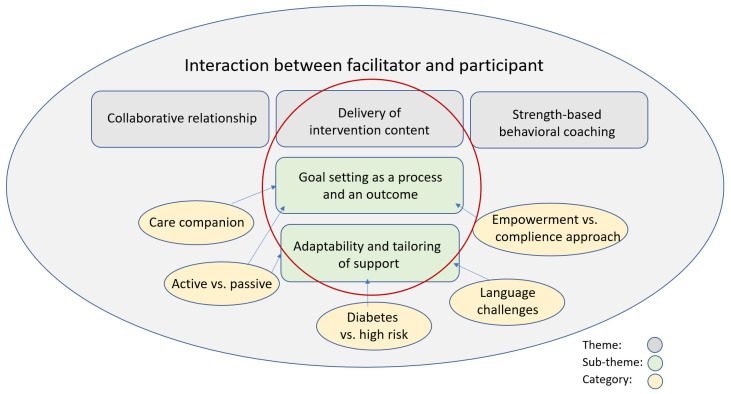
Thematic map showing themes and subthemes developed from the data.

Thematic analysis was carried out following the steps suggested by Braun and Clark (2006) [44]. The process of analysis began in the data collection phase during note taking by one of the moderators (L.T.) who led the discussions. To immerse in and familiarize with the data, the recordings were listened to and the transcripts read thoroughly several times. The data were considered to have enough information power for conducting a rigorous analysis [45]. The dataset was coded and categorized using the software program NVivo 10. The author (L.T.) coded the transcripts. The coding and theme development was discussed with the last author (H.M.A.), who is an experienced qualitative researcher. Agreement on theme labels/description was achieved with all authors. The developed themes were further revised and thematic maps developed (Figure 4 in the qualitative results section is one example).

## 3. Results

### 3.1. Quantifying the Interaction

The EFA resulted in three factors: (1) Collaborative relationship, (2) Delivery of intervention content and (3) Strength-based behavior coaching. Cronbach’s alpha of 0.86, factor 1= 0.89, factor 2 = 0.83 and factor 3 = 0.74 indicated that the internal consistency of the factors was good/acceptable, i.e., the items in each factor are related (Table 2). Factor 1 explains 57% of the variance in this 14-item scale; factor 2, 19%; and factor 3, 15%, respectively.

The Likert summated scores showed values from 75–81 for the three factors, indicating that the intervention was delivered as intended. When comparing the summated scores between facilitators, a significant difference (*p* = 0.0017) was found in factor 2, Delivery of intervention content (Table 3). This indicates that the facilitators delivered the intervention inconsistently with regards to the items in that factor, with facilitator 1 (F1) showing the highest and facilitator (F2) the lowest fidelity of delivery (Table 3). Differences between the facilitators were found due to the use of the session guide (6.7) and the demonstration of knowledge pertaining to the content of the session (6.6).

Participant engagement, measured by percentage of time the participant talked, showed that the participants talked on average 40% of the sessions. No significant difference between facilitators was found. There was a slight increase in time the participant talked with increasing number of sessions, but this was not consistent.

### 3.2. Qualifying the Interaction

The three constructs from the quantitative results were deductively identified as themes in the qualitative data: (1) Collaborative relationship, (2) Delivery of intervention content and (3) Strength-based behavioral coaching. In addition, other themes were developed inductively. These themes were categorized as subthemes to each predefined theme. All themes are connected to aspects of interaction between the facilitator and participant (Figure 4).

In line with the quantitative results, the two subthemes “Goal setting as a process and an outcome” and “Adaptability and tailoring of support” also showed differences in the delivery of the intervention. To illustrate these two themes, extracts from the data in the form of quotes from the facilitators are presented below (F: Facilitator, M: Moderator).

#### 3.2.1. Goal Setting as a Process and an Outcome

The facilitators’ experience of supporting participants in the goal setting process for behavior change varied between the facilitators. For some of them, goal setting was a central part of the intervention delivery leading to a more directive approach, while for others, the process of goal setting was less transparent:

*F1: No, I think I write down a lot of what they say like: I want to, or I have a problem with this, or I never eat lunch or something. And I put that in and then I can go back and read that. Yes, so in some way they have issues, but it is not goals, really. But yes, I try to go back and discuss what they said to me.*

*M: So, you don’t mention goals?*

*F2: I definitely write down the goal, for example this client was walking and I asked: Have you been continuing walking and I mention it every ten days, asking specifically what he is doing.*

*M: Do you think that is important to be specific?*

*F4: So, I think it is very good to be specific because they have so many stories as well. At the end of the day, you’ll get a long list, they will have done everything in the manual, but you will not have the specific goals right? So, I think that it is good to put them in, the specific goals.*

The facilitators encouraged the participants to seek support from a care companion defined as a person in the participants’ close network, such as a partner, other family member or a close friend. The goals were supposed to be decided in collaboration with the care companions following the SMART2D intervention guide. When facilitators experienced difficulties in communicating the benefits of setting goals in collaboration with the care companion, they used different strategies to involve the care companions in the process. One applied strategy was to include the care companion through setting a clear goal during the session and encouraging the participant to discuss the goal with the care companion afterward. Below is an example of a situation with the care companion actively involved in the goal setting using a more directive approach:

*F2: So, I told them: Hey, so, an example of things that you can do for activities would be that you identify healthy recipes to cook and then he says: “Yes, but my wife is the one who cooks, she is my [participant’s] care companion.” She was there, he talked to her that day. “But yes, that is something we can do, yes, I can see something healthy and then tell her.” It seems like she is around when he is talking to me. And they are very active, so I suggested goals, then he said: “Yes, ok, this is something I can do.” And then I wrote it down as a goal.*

Concerns were expressed about the involvement of the care companion in goal setting by all facilitators. Many of the participants seemed to be satisfied with having the facilitator as their social support component, and they expressed no need for an additional care companion. There were also examples, although not many, where the social support from family members or friends as care companions worked well, according to the facilitators:

*F1: Well I have one couple who sit down and go through and are super prepared when I talk to them. But I would say that they are maybe an exception…*

*F4: Yes, and then there is one who has a friend and a daughter so there are three of them who discuss and do things with them. So, you know these are a few of the success stories that I see. And you can see that …they have specific goals and they decide yes, I will do the steps and I promise, I hope that I can do like five thousand steps every day, that’s my goal, we will see if I can achieve that. So, I’ve seen those.*

To maintain contact with the care companion, it was clearly seen as an advantage if the persons lived together, although the role was then more part of everyday conversations:

*F3: The best chance of anything happening is that they live together. But it seems much more informal. “Yes, yes we discussed it”, there is no, “we sat down and we talked about our goals”. That just doesn’t happen, even in the ones for me who are working, who are living together.*

To identify measurable behaviors, activities were both suggested by the facilitators and built on suggestions from the participants. The facilitators suggested ideas and gave participants time to reflect on them. This was expressed as a strategy to encourage the participants to make goals on their own, in line with the empowerment approach:

*F4: Like for my participants for example I suggest, so how about, I mean have you thought about doing this, doing that, and they are like yes, I have tried that. One participant said that she has tried standing one leg while brushing her teeth and God, that doesn’t work for her because she doesn’t have balance but she decided to stand while talking on the phone. And then, that is just one aspect on top of the walks that she has decided she is going to make, which is great. So, she decided she could, she tried, yes, she tried. But she decided she will do the standing while talking on the phone and then she will take some walks with her husband every day at least for 30 min, and then she will decide, I mean, they will try to increase the time they do the walking. […] This is the thing when you have a situation for them to reflect and that is the situation I’m talking about. So, when they start reflecting upon it then you know an idea pops up and it becomes a goal.*

Goal setting was talked about during several calls and was perceived as more important for the less active participants and less important for already active participants:

*F3: But I’m thinking in general if we have someone who is already very, very physically active and we pass that message on, but in terms of goals, there is no need for particular goals! If I talk to a person who is walking, I don’t know, 15,000 steps a day and who is very active, and he is playing bandy and he is playing this and he is playing that. I don’t think I need to convince him during that particular session. Then I need to inform him of the content of the session and the message of the session. But to then try to get someone who is already very active to start walking upstairs if he already is… it is not my idea of an achievement.*

#### 3.2.2. Adaptability and Tailoring of Support

All facilitators emphasized the need to tailor the intervention due to the participants’ needs session by session to each participant. The sessions also needed to be delivered at different times of the day to suit the participants’ schedules. The main difference was how the sessions were delivered between participants with the T2D diagnosis versus persons at risk of developing diabetes. Participants with diabetes were already familiar with advice on the suggested lifestyle modifications on diet and physical activity:

*F1: Yes, and they have a lot of things going on… yesterday I talked to someone about “nyckelhålet” [Swedish healthy food label] and she told me: “Yes, when I got my diagnosis, like when was it was one year ago, I had a dietician talk or help, lecturing me about “nyckelhålet” and stuff like that so I know that already. A lot of my people have this.”*

The facilitators talked about active versus nonactive interventions depending on the activity level of the participants. The already active participants seemed easier to engage in the intervention activities. A need for social contact among many participants was recognized, and sometimes the facilitators considered themselves to function as a more general social support in addition to support on T2D prevention and management. To have someone to discuss concerns with was useful for maintaining motivation. The facilitators described that they had a personal relationship with the participants and that some participants seemed to be lonely and having a lot of concerns:

*F3: I was concerned that it would not be enough or that it would you know… That was not met. People were very happy, and the conversations can go on for as long as you want. I think that I was not expecting this personal, personal relationship to the development which is also what leads me to think that what are we actually delivering? We are delivering this package, but I think for a lot of people it is more than that. Or even other than that, there is this very strong social support factor that I feel that I’m delivering. Not only for diabetes, but for health. Because one person has called me “her personal health”. And I say: Yes, we are here to facilitate but the main thing is to support you in to working with someone else. But also, the fact that people are very lonely and do have a lot of concerns. This is not just a delivery, this is more.*

Language was a challenge in that Swedish was not the first language of many participants. Easy Swedish, a form of Swedish without technical and complicated medical terms, was used to deliver the intervention to participants with limited Swedish language skills. When the participants had difficulties in expressing themselves, they could suggest taking questions in English. No participants preferred to use interpreters, although this could have been arranged and was offered. Instead, in some cases, the participant had help from relatives or spouses to participate in the program because of limited language skills in Swedish or English. In addition, some of the facilitators had other languages than Swedish as their first language, and they expressed that it was easier when using one’s first language in the delivery of the intervention:

*F2: And obviously the sessions that I do in Spanish feel more comfortable because it feels that it has a better flow. In terms of when they say something for example, sometimes the participants in Swedish just say something and stop and then it’s like they don’t want to continue talking about it, while the participants in Spanish, they just want to go on and on about one specific little thing.*

## 4. Discussion

In this study, we used mixed methods to evaluate and analyze interaction as part of the fidelity of a telephone-facilitated health coaching intervention among participants at high risk of T2D or already diagnosed with T2D. The interaction between facilitators and participants is central in the communication. The aim of the health coaching sessions was not simply to deliver information and improve knowledge but to also achieve small changes in diet and physical activity to improve self-management for diabetes care and prevention.

Fidelity could be confirmed for the delivery of the intervention in terms of intervention content, utilization of strength-based behavioral coaching techniques and formation of a collaborative relationship. Yet differences were found between the facilitators in terms of delivery of intervention. The qualitative data gave further insights to the difference in the delivery of intervention by exploring how the coaching approaches adopted by the facilitators differed. Differences in the delivery were particularly apparent in two qualitative subthemes: goal setting as a process and an outcome and adaptability and tailoring of support. As such, the qualitative findings deepened the understanding of fidelity of the implementation from facilitator viewpoint, and we could find potential explanations about why they did or did not follow some aspects of the protocol.

Both fidelity and adaptation are essential to consider when implementing preventive interventions [46]. Moreover, from the fidelity aspect of this study, it was necessary to balance the fidelity with adaptation needs, as acknowledged in the literature on intervention fidelity [29,46]. The facilitators had to balance the extent to which they followed the intervention guide with tailoring of support to match the participants’ needs. This can be a dilemma, since the adaptation of an intervention is often needed when it is implemented in real-life settings, although the tailoring risks weaken the fidelity [29]. To modify the intervention guide to be more flexible in allowing more tailoring would potentially increase the adherence of the intervention delivery.

Goal setting was a difficult process. The qualitative results revealed differences in how goal setting was viewed between the facilitators and their different delivery styles, top–down versus bottom–up. We observed differences between the facilitators. Some used more of a compliance-based approach [20] by giving suggestions of goals that the facilitator wanted the participant to apply, while others used an empowerment approach where the participant was encouraged to set his/her own goals. The latter is in line with the empowerment approach described by Funnell et al. (2005), where the control of self-management is handed over to the patient [20]. This way of empowering was used by some of the facilitators in our study.

Both goal setting with an empowerment approach as well a more directive and compliance-based approach could be noted among the facilitators. The difference in goal setting style between facilitators could mean that the training was insufficient to enable a uniform approach in terms of empowerment and that the training period of facilitators might have been too short. More training in specific psychological techniques such as MI [23] or strength-based behavior coaching [22] may have been beneficial for the facilitators. At the same time, we cannot assume that a more uniform approach is necessary for the delivery of the intervention itself, although it would have strengthened the fidelity. The language barriers mentioned as a hinderance in the delivery could also mean that it takes more skills from the facilitator to guide a person with poorer verbal interaction skills in an empowering way.

In particular, goal setting was complicated to achieve when it was supposed to happen in collaboration with the care companion. The facilitators reported a low interest and difficulties with involving a care companion in adapting to a healthier lifestyle. Lack of time was also mentioned as a hinderance. This could be one of the reasons why the implementation of the care companion concept was challenging for the SMART2D project in the Swedish context. Evidence shows that peer support has positive effects on behavior change [47,48,49]. The facilitators were both the participant’s personal health coach and someone to talk with as a relief from loneliness. This is an example of socioemotional patient-centered interaction in terms of the therapeutic relationship [50], in line with the collaborative spirit of MI [23] and the relational function of interactions [26].

To mitigate loneliness, relationship preferences as well as characteristics of personal relationships should be considered [51]. Although the facilitators served as a social support, to establish a care companion relationship with someone in the participants’ close network is a more sustainable solution. The facilitators were able to provide support only during the intervention period and were not present in participants’ daily lives. We found a few positive examples of a person in the participants’ close network such as a spouse, daughter or friend functioning well as a care companion.

The facilitators emphasized the need for tailoring the sessions to participants’ needs. This study showed that the participants who had T2D had previous knowledge about lifestyle modification compared with participants at high risk of developing diabetes but no manifest diabetes. Thus, this might require different content in the counseling sessions compared to more basic information needed for the persons that are not receiving counseling. The focus would be on what to change and why for persons at high risk of developing diabetes prior to working on how to operationalize the change process. Another difference in the delivery was recognized in communication related to language skills. Although interpreters were offered and facilitators with multiple language skills were used, language skills were discussed as a barrier, as low language skills hindered the flow of intervention delivery. This study underlined the gains of using language-skilled facilitators to minimize barriers in the intervention delivery.

## 5. Strengths and Limitations

The mixed methods design is a strength, as it allows triangulation of methods and results and generates more comprehensive and accurate fidelity findings [29]. In this study, we examined intervention delivery from two perspectives: the quantitative data found differences between facilitators in delivery of the intervention, and qualitative data confirmed the findings and provided potential explanations. In this way, we had the opportunity to learn more about why the facilitators did or did not follow some parts of the intervention protocol. This knowledge is valuable from the fidelity aspect. Furthermore, the recording of sessions allowed us to have two independent raters who had the opportunity to listen to each recording several times. Research shows that there are several advantages to using audio recordings in the evaluation of intervention fidelity, as recordings are less intrusive and inexpensive compared with real-time observations and are also less likely to influence participants [52].

The EFA allowed us to test our instrument and build factors from the items included. The analysis revealed three factors based on 14 items with 9 items removed from the final analysis. Three items had missing values, and the additional four statements were removed due to cross-loading, i.e., the items were too alike to separate in the analysis or due to low factor loadings, indicating the items did not contribute to the analysis of that factor. The summated Likert scores allowed us to investigate if there were differences between the facilitators in the three factors but did not allow us to separate the items in each factor. Instead, the qualitative analysis was used as a measure to explore why there were differences in one of the factors.

A number of limitations exist in this study. One is that sessions conducted by the skilled language facilitators in Somali and Arabic were excluded from the analysis, and therefore, the full sample is not represented. Additionally, the analysis of the interaction tool showed scoring differences between the two raters, and although small, the differences had large effects on the intraclass correlation coefficient and subsequently the reliability of the consistency of the tool. Further, although we had richness of data, the quantitative sample was small. Another limitation is that the qualitative data are only from the perspective of the facilitators. Therefore, interviews with participants would have been valuable to obtain a full picture of the challenges and potential improvements for this type of intervention.

## 6. Conclusions

Evaluating the interaction between facilitators and participants is an important aspect of intervention fidelity. In this telephone-facilitated health coaching intervention, differences were found between facilitators in goal setting and tailoring of the intervention delivery. Clear guidelines and training of facilitators in specific techniques are required for a more uniform delivery approach in the empowering of participants, in particular for goal setting. Nevertheless, the intervention guide should be flexible and allow adaptations to participants’ needs. Tailoring of an intervention is necessary, and language-skilled facilitators are needed to minimize barriers in intervention delivery. To learn from processes in intervention, implementation is important for self-management and prevention of T2D.

## Figures and Tables

**Table 1:** Telephone-facilitated health coaching sessions included in the Swedish SMART2D intervention.

Session	Title	Content
1	Introductory session	Getting to know the program. Why work with a care companion to make lifestyle changes?
2	Increase physical activity in daily life and reduce sedentary lifestyle	The importance of physical activity and how this can be increased in daily life
3	Healthy eating: Regular, balanced and healthy	The importance of regular, balanced and healthy meals
4	Physical activity through the life course	Discussion on how physical activity levels have changed over the years
5	Fruit and vegetables	The importance of eating fruit and vegetables every day
6	Increasing your daily physical activity	Discussion on current situation and potential possibilities for improvements
7	Sugar	How sugar consumption can be decreased in daily life
8	Finding a physical activity that suits you	Discussion of options/choices to physical activity
9	Healthy lifestyle—moving forward	How has it been to try to change to a healthier lifestyle and how can this be maintained?

**Table 2:** Factor score distribution for the final domains (Likert summated scores).

		Factor 1	Factor 2	Factor 3
Statement		Collaborative Relationship	Delivery of Intervention Content	Strength-Based Behavioral Coaching
5.4	The session holds a positive atmosphere	0.97		
5.2	The participant engages with the facilitator during the session	0.89		
5.1	The participant shows interest in the session	0.83		
4.1	The facilitator works with the participant to identify his/her strengths	0.58		
6.7	The facilitator used the session guide to conduct the session		0.83	
6.3	The facilitator encouraged the participant to work on the sessions specific suggested tasks		0.69	
6.4	The facilitator encouraged the participant to set goals		0.64	
6.1	The facilitator informed the participant about the content of the session		0.60	
6.6	The facilitator demonstrated knowledge pertaining to the content of the session		0.54	
6.9	The facilitator gave the participant the opportunity to ask questions			0.85
4.4	The facilitator gave sufficient time for participant reflection and response			0.74
5.6	The participant shares experiences with the facilitator			0.56
4.3	The facilitator verbally reflects on participant’s talk			0.49
4.5	The facilitator summarizes participant’s talk			0.44
	Eigenvalue	5.0	1.7	1.3
	Variance explained	57%	19%	15%
	Cronbach’s alpha	0.89	0.83	0.74

**Table 3:** Factor score distribution (median and interquartile range *) for the final domains.

	Collaborative Relationship	Delivery of Intervention Content	Strength-Based Behavioral Coaching
	Median (IQR)	Median (IQR)	Median (IQR)
Total (*n* = 40)	75 (69–81)	81 (76–94)	75 (70–78)
Comparison between facilitators (F)	75 (75–81)	94 (85–100)	76 (70–80)
F1 (*n* = 10)			
F2 (*n* = 10)	75 (59–75)	75 (73–75)	70 (65–75)
F3 (*n* = 12)	78 (72–97)	81 (79–92)	75 (71–80)
F4 (*n*= 8)	78 (70–84)	81 (78–90)	73 (70–75)
*p*-value	0.1465	0.0017 *	0.2079

* The sum score was standardized to range from 0 to 100.

## Data Availability

All relevant data are within the paper. Additional data from the interaction scoring are available from the authors upon reasonable request.
